# Coexistence of Neonatal Lupus Erythematous and Sturge–Weber Syndrome

**DOI:** 10.1155/2021/3616429

**Published:** 2021-12-30

**Authors:** Zahra Nikyar, Parvaneh Hatami, Zeinab Aryanian, Kambiz Kamyab Hesari, Azadeh Goodarzi, Anahita Borzouei

**Affiliations:** ^1^Autoimmune Bullous Diseases Research Center, Tehran University of Medical Sciences, Tehran, Iran; ^2^Department of Dermatology, School of Medicine, Razi Hospital, Tehran University of Medical Sciences, Tehran, Iran; ^3^Department of Dermatology, Babol University of Medical Sciences, Babol, Iran; ^4^Department of Dermatopathology, School of Medicine Razi Hospital, Tehran University of Medical Sciences, Tehran, Iran; ^5^Department of Dermatology, School of Medicine, Rasoul Akram Hospital, Iran University of Medical Sciences, Tehran, Iran; ^6^Department of Dermatology, Mashhad University of Medical Sciences, Mashhad, Iran

## Abstract

Neonatal lupus erythematous (NLE) is a rare condition presented by lupus dermatitis shortly after birth or later following sun exposure. Sturge–Weber syndrome (SWS) is also an uncommon congenital condition characterized by extensive capillary malformation and ophthalmic and/or neurologic involvement. Here, we describe the first case of coexistence of NLE and SWS which posed a significant diagnostic challenge to clinicians.

## 1. Introduction

Neonatal lupus erythematous (NLE) is a rare condition produced by the transplacental passage of maternal autoantibodies [1]. NLE usually presented with lupus dermatitis including annular erythemato-squamous plaques developing shortly after birth or later following sun exposure [2].

The skin lesions often resolve spontaneously with or without leaving hypopigmented or hyperpigmented scar within the first year of life.

Sturge–Weber syndrome (SWS) is also an uncommon congenital condition characterized by extensive capillary malformation and ophthalmic and/or neurologic involvement [3].

Here, we describe a case that looks worthy of record since it presented with both NLE and SWS which has not been previously reported.

## 2. Case Report

**Figure 1:**
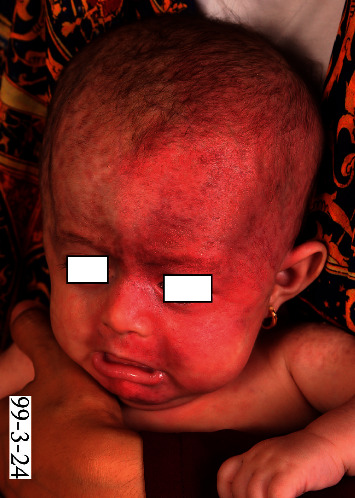
Extensive port-wine stain since birth.

A 3-month-old girl, born to nonconsanguineous parents, presented to our clinic with extensive port-wine stain on the left facial area since birth (Figure 1).

She was the full-term production of an uncomplicated pregnancy to a healthy mother without a history of abortion or any known medical condition.

**Figure 2:**
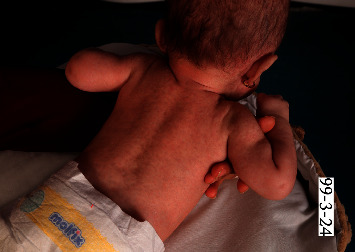
Multiple erythematous/hyperpigmented macules.

On physical examination, the patient had a generally good condition. An extensive port-wine stain was noted distributing on V1, V2, and V3 areas on the left side of the face without involvement of mucosa. Multiple atrophic and hyperpigmented patches with telangiectasia on both sides of the face were seen which were more prominent on the forehead and temporal region and around the eyes. They presented since birth and were more obvious on the right side of the face due to presence of the port-wine stain on the other side. Multiple erythematous and/or hyperpigmented punched-out lesions, some with scaly appearance and/or atrophic surface, were noted on the trunk and proximal parts of extremities (Figure 2).

Initially, it was assumed to be a case of SWS and the patient was referred to the pediatric department for further ophthalmologic and neurologic examinations regarding presence of any abnormality in favor of SWS. No remarkable finding was noted in evaluations, except for bilateral benign horn cyst found in brain sonography.

**Figure 3:**
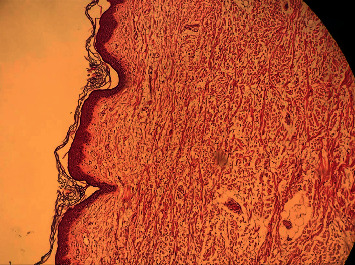
Dermal infiltration of spindle cells in a collagenous stroma containing abundant mucin intermixed with some mast cells.

**Figure 4:**
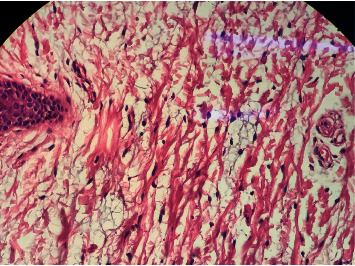
A magnified view of histopathological changes seen in the patient's tissue sample.

However, we did not have any explanation for her atrophic punched-out lesions in the setting of SWS. Therefore, histiocytosis, mastocytosis, Goltz syndrome, and scar formation following maternal viral or parasitic infections during pregnancy including TORCH infections (toxoplasmosis, rubella, cytomegalovirus, and herpes simplex) were considered as the most relevant differential diagnoses, despite the absence of any applicable maternal history. A 4 mm punch biopsy was taken from one of the atrophic lesions of her trunk. Histopathologic assessment showed dermal infiltration of spindle cells in a collagenous stroma containing abundant mucin intermixed with some mast cells which was more compatible with “progressive mucinous histiocytosis” (Figures 3 and 4). “Cutaneous mucinosis of infancy” and “self-healing juvenile cutaneous mucinosis” were also suggested as differential diagnoses.

However, clinical manifestation of these disorders was quite different from that seen in our case.

Moreover, IHC study for CD 31, S100, C-kit, and lysozyme was performed which was not diagnostic for any of these diagnoses.

Unfortunately, we could not perform serologic evaluation for TORCH infections because of some financial issues.

Considering clinical appearance of the lesions including atrophy and telangiectasia and existence of abundant mucin in histopathologic assessment which has been reported as a rare finding in patients with lupus erythematosus [4], a diagnosis of NLE was suspected and relevant laboratory data were requested. Serologic examination of sera of the patient and her mother revealed the presence of anti-Ro/SS-A and anti-La/SS-B in high values in both and FANA positivity in her mother (titer: 1/3200).

Fortunately, any heart block or other cardiologic abnormalities were not found by the pediatric cardiologist, and other laboratory evaluations were within the normal range.

These data confirmed the diagnosis of NLE without internal involvement for our patient.

Considering the fact that extensive capillary malformation was previously reported as a rare manifestation of NLE [3] and also normal ophthalmologic and neurologic evaluations in our patient, the diagnosis of SWS seemed to be unlikely and port-wine stain was assumed to be an uncommon feature of her NLE.

Surprisingly, she developed an episode of nonfebrile tonic-clonic seizure about one month later. Hence, she was admitted and received phenytoin, phenobarbital, and Tegretol. Brain CT scan revealed mild subdural hematoma and streaky calcification compatible with leptomeningeal angiomatosis which, in context of having an extensive port-wine stain, returned us to the diagnosis of SWS. A secondary ophthalmologic consultation was requested in which glaucoma was noted in the left eye and the diagnosis was even more confirmed.

Then, the patient was discharged on phenobarbital 15 mg/BD and syrup Tegretol 60 mg/BD.

The patient remained seizure-free until the 10^th^ month of her life when the second episode of tonic-clonic seizure happened, and her medication was changed to phenobarbital, clonazepam, and aspirin. A brain MRI was performed which revealed leptomeningeal enhancement and hypotrophy in the left cerebral hemisphere indicative of SWS.

**Figure 5:**
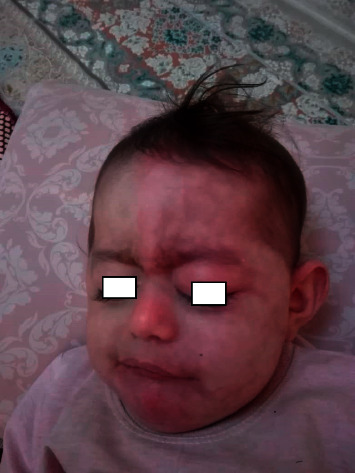
Remarkable resolution of port-wine stain following PDL therapy.

**Figure 6:**
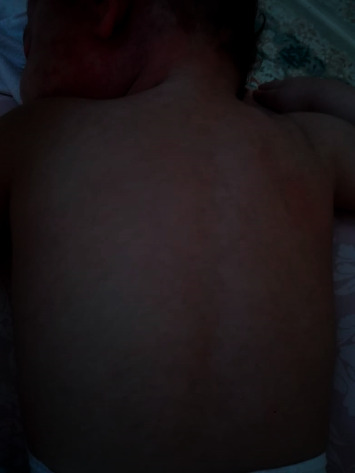
Spontaneous resolution of cutaneous lesions of NLE.

Now, the child is 15 months old. No seizure occurred within the last 5 months. Her port-wine stain considerably improved following 4 sessions of PDL (pulsed-dye laser) therapy and other cutaneous lesions getting better spontaneously (Figures 5 and 6).

## 3. Discussion

We reported a case of NLE and SWS where both of them are considered as rare conditions. To the best of our knowledge, the coexistence of these disorders has not been reported until now.

NLE is an uncommon disorder caused by transplacental transfer of maternal anti-Ro and anti-La autoantibodies. The most common manifestation of NLE is cutaneous lesions developed in 50% of cases, and they usually appear in the first months of life [5] but can be noted since birth in a minority of cases, such as ours.

In our patient, most of the lesions presented as atrophic and telangiectatic macules on the scalp and trunk which is more likely representative of occurrence of inflammatory phase during pregnancy. This might explain the absence of distinct inflammatory features of LE on histopathologic evaluation. In fact, the absence of typical annular lesions of NLE, as well as unspecific histopathologic findings in this case, indicates the importance of having clinical suspicion for reaching correct diagnosis.

It is worthy to note that the presence of abundant mucin in histopathologic assessment might be a feature of NLE lesions [5] and should not mislead us to more common differential diagnoses such as different types of mucinosis in case of clinical suspicion to LE.

Widespread cutaneous lesions of NLE at birth are very rare and might be considered as a proof for the hypothesis that UV radiation, despite previous reports [6], might not have an essential role in the expression of intranuclear antigen and developing clinical lesions.

Another interesting feature of our case was the fact that her mother initially seemed to be healthy, but a more detailed history revealed that she had arthralgia and hence was referred to the rheumatology department where it turned out that she had some criteria of SLE.

SWS is also a rare syndrome characterized with facial angioma reaching territory V1, ophthalmologic anomaly (mostly congenital glaucoma), and neurological malformation, such as leptomeningeal angioma might lead to seizure or neurological deficit [2]. SWS is classified into 3 types: type 1 (complete or classic), type 2 (bisymptomatic), and type 3 (rough form) [7].

Our case was initially presented by facial port-wine stain which could be considered as an uncommon cutaneous presentation of NLE considering normal first ophthalmologic and neurologic evaluation. However, occurrence of subsequent seizure episodes and detecting glaucoma on further assessments confirmed the diagnosis of SWS with complete manifestation (type 1).

As mentioned above, nothing abnormal was found on the first ophthalmologic assessment of our patient which might be due to developing glaucoma later, during the course of the disease.

Even though the most common form of ocular complication in SWS is congenital glaucoma, it can also develop during childhood or even later in life [8] which shows the significance of careful ophthalmic follow-up of these patients.

In conclusion, we report an extraordinary case of NLE not only for having lesions since birth and with lack of maternal history which posed a significant diagnostic challenge but also for simultaneously having another rare condition (SWS) which made it even more challenging to diagnosis.

Making correct diagnosis in this case was important in several aspects: firstly, diagnosis of each of these disorders necessitates more systemic evaluations which led to detection of glaucoma in our case. Another important issue for patients with NLE is a relatively higher risk of developing other autoimmune disorders such as rheumatoid arthritis and Hashimoto thyroiditis during their childhood. Hence, they need long-term follow-up even though they experience spontaneous resolution of their extra cardiac manifestations [9].

Moreover, diagnosis of NLE in this case led to detection of SLE in her mother which was previously neglected. This also mandates strict follow-up of her mother during subsequent pregnancies due to higher risk of complete heart block in neonates which can be prevented by using IVIG and hydroxychloroquine early during pregnancy [10–14].

Therefore, careful examination and clinical suspicion besides appropriate paraclinical assessment are the key factors for correct diagnosis and management of patients.

## Data Availability

Data are available on reasonable request to the corresponding authors.
